# Pneumatosis intestinalis and portal venous gas secondary to Gefitinib therapy for lung adenocarcinoma

**DOI:** 10.1186/1471-2407-12-87

**Published:** 2012-03-10

**Authors:** Joo Young Lee, Hye-Suk Han, Sung-Nam Lim, Young Kwang Shim, Yong Hyeok Choi, Ok-Jun Lee, Ki Hyeong Lee, Seung Taik Kim

**Affiliations:** 1 Department of Internal Medicine College of Medicine, Chungbuk National University, 410 Seongbong-ro, Heungduk-Gu, Cheongju 361-711, South Korea; 2 Department of Pathology, College of Medicine, Chungbuk National University, Cheongju, South Korea

## Abstract

**Background:**

Pneumatosis intestinalis (PI), defined as the presence of gas in the bowel wall, and portal venous gas (PVG) are relatively rare radiological findings. Although several chemotherapeutic agents and anti-vascular endothelial growth factor agents are reported to be associated with PI and PVG, an association with anti-epidermal growth factor receptor (EGFR) agents has not been described previously.

**Case presentation:**

The present report describes a case of PI and PVG secondary to treatment with an EGFR tyrosine kinase inhibitor. A 66-year-old woman who had been diagnosed with metastatic lung adenocarcinoma presented with nausea, vomiting and abdominal distension after commencing gefitinib. A computed tomography (CT) scan of the abdomen revealed PI extending from the ascending colon to the rectum, hepatic PVG, and infarction of the liver. Gefitinib therapy was discontinued immediately and the patient was managed conservatively. A follow-up CT scan 2 weeks later revealed that the PI and hepatic PVG had completely resolved.

**Conclusion:**

This is the first report of PI and PVG caused by EGFR tyrosine kinase inhibitor. Although these complications are extremely rare, clinicians should be aware of the risk of PI and PVG in patients undergoing targeted molecular therapy.

## Background

Recent advances in our understanding of the biology and molecular mechanisms of cancer have led to the introduction of molecular-targeted agents for the treatment of non-small cell lung cancer (NSCLC). Gefitinib is an orally active selective inhibitor of the epidermal growth factor receptor (EGFR) tyrosine kinase, an enzyme that regulates the intracellular signaling pathways implicated in the proliferation and survival of cancer cells [1]. Somatic mutations in the region of *EGFR *that encodes the tyrosine kinase domain of the receptor have been identified in patients with NSCLC and many studies report that NSCLC patients who carry these mutations are highly responsive to gefitinib [2,3].

In general, targeted molecular therapies such as gefitinib have good toxicity profiles. However, some patients develop specific and severe toxicities, since these molecular targets are also expressed in normal cells. Although gefitinib is generally well tolerated, its most commonly reported side effects are of the gastrointestinal tract (diarrhea, nausea and vomiting) and skin (rash, acne, dry skin and pruritus). Severe gastrointestinal toxicity secondary to gefitinib is uncommon, and only 1% of patients treated with gefitinib develop grade 3 or 4 diarrhea [3].

The present report describes the development of pneumatosis intestinalis (PI) and portal venous gas (PVG) in a patient with metastatic lung adenocarcinoma who had received gefitinib therapy.

## Case presentation

**Figure 1:**
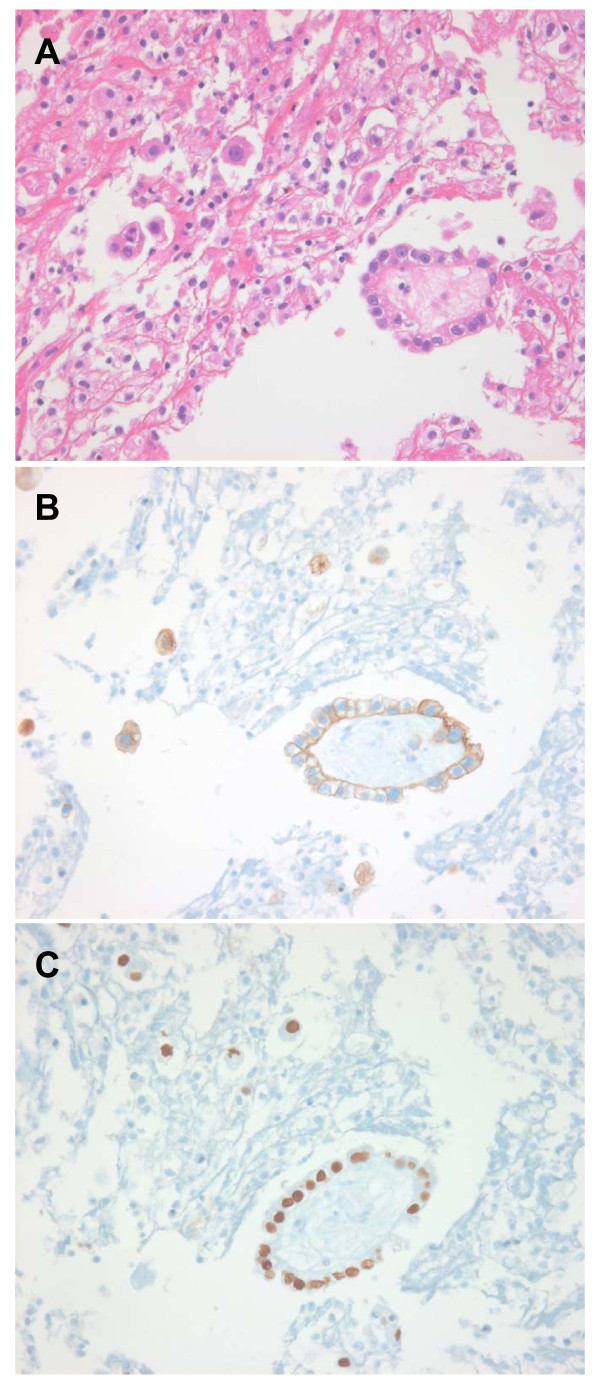
**Pathological features of a cell block from the malignant pleural effusion**. Histological examination of the cell block revealed metastatic adenocarcinoma (A, H&E × 400). Immunohistochemistry showed that the tumor cells were positive for (B) cytokeratin 7 and (C) thyroid transcription factor-1 (× 400).

A 66-year-old woman was diagnosed with lung adenocarcinoma with malignant pleural effusion in December 2009. A computed tomography (CT) scan of the chest at diagnosis revealed a mass in the right middle lobe and a right-sided pleural effusion. Histological examination of a cell block from the pleural effusion following hematoxylin and eosin staining confirmed a diagnosis of metastatic lung adenocarcinoma. Immunohistochemical staining showed that the tumor cells were positive for cytokeratin 7 (1:600; NeoMarkers, California, USA) and thyroid transcription factor-1 (1:300; Leica, Newcastle-upon-Tyne, UK), but negative for cytokeratin 20 (1:200; Leica) (Figure 1). The patient received six cycles of palliative chemotherapy, consisting of gemcitabine and cisplatin, which resulted in a partial response. In November, 2010, the patient developed anorexia and abdominal distension. A CT scan of the chest revealed that the disease had progressed. Moreover, a CT scan of the abdomen revealed mild ascites, omental dirty fat infiltration, and lymph node enlargement in the porta hepatis, peripancreatic, interaortocaval and paraaortic areas. Histological examination of a cell block from the ascites confirmed a diagnosis of metastatic adenocarcinoma, and the immunohistochemical profile was consistent with that obtained previously for the malignant pleural effusion. To exclude the existence of another primary site, esophagogastroduodenoscopy, colonoscopy and positron emission tomography-CT scanning were performed; no intra-abdominal primary site was identified. On the basis of these findings, a diagnosis of primary lung adenocarcinoma with peritoneal metastases was assigned. The tumor tissue was analyzed for the presence of an *EGFR *mutation using the PNAClamp™ EGFR Mutation Detection kit (PANAGENE, INC. Daejeon, Korea). In the cell blocks from the pleural fluid and the ascites, an L858R mutation in exon 21 was detected. Gefitinib therapy was therefore commenced.

**Figure 2:**
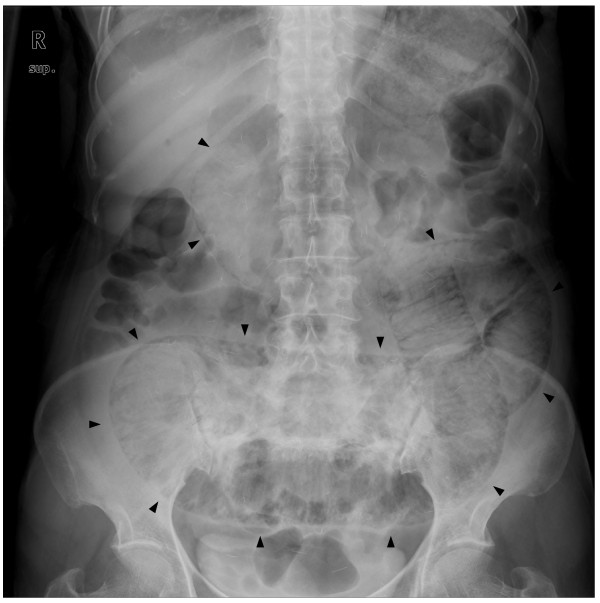
**A plain abdominal radiograph**. Abdominal X-ray showed a dilated colon and the presence of intraluminal air along the entire wall of the colon (black arrows).

**Figure 3:**
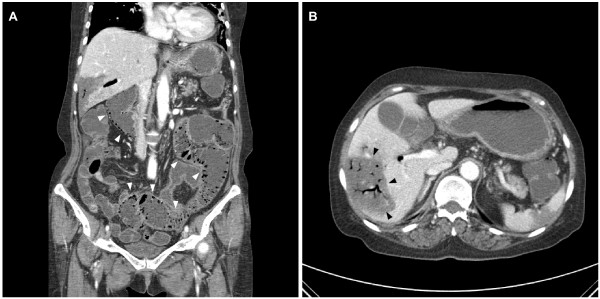
**Computed tomography (CT) image of the abdomen and pelvis**. The CT scan shows extensive bubble-like pneumatosis intestinalis along the entire colon (A, white arrows), branching lucencies indicating hepatic portal venous gas, and a low-attenuation lesion in the right lobe of the liver, suggestive of infarction of the liver (B, black arrows).

In January, 2011 the patient presented with a 7-day history of progressively worsening nausea, vomiting, diarrhea and abdominal distension. On examination, she appeared non-toxic and was afebrile with stable vital signs. Although abdominal distension with decreased bowel sounds and generalized mild abdominal tenderness upon palpation were detected, no peritoneal signs were present. Investigation of blood chemistry revealed an alanine aminotransferase of 1435 IU/L, an aspartate aminotransferase of 2178 IU/L, a total bilirubin of 0.85 mg/dL, a blood urea nitrogen of 36.1 mg/dL and a creatinine of 1.38 mg/dL. A stool culture was negative. A plain abdominal radiograph showed a dilated colon with diffuse and extensive intraluminal air (Figure 2). A CT scan of the chest and abdomen revealed that the size of the mass in the right middle lobe, the volume of the malignant pleural effusion, the volume of the ascites and the level of omental dirty fat infiltration had all markedly decreased. However, PI extending from the ascending colon to the rectum was detected in the absence of pneumoperitoneum or other abnormalities (Figure 3A). Moreover, a large volume of hepatic PVG and a low-attenuation lesion in the right lobe of the liver were observed. These findings were considered to be suggestive of an infarction of the liver (Figure 3B). Although the CT images were striking, surgical exploration was not performed since the patient was non-toxic and had mild symptoms. The patient was not taking any medicine except gefitinib. Gefitinib therapy was immediately discontinued and the patient was managed conservatively with nasogastric-tube-drainage and prophylactic antibiotics. Two weeks later, the gastrointestinal symptoms improved and a reduction in the levels of the liver enzymes was observed. A follow-up CT scan at this time-point revealed that the PI and HPVG had completely resolved.

Although gefitinib had led to a partial response, this therapy was now considered to be contraindicated in this patient. Pemetrexed was therefore commenced instead as a third-line therapy. However, the malignant pleural effusion and peritoneal carcinomatosis became exacerbated, and the patient died of bacterial pneumonia with sepsis 18 months after the initial diagnosis.

## Conclusions

PI is defined as the presence of gas within the bowel wall and represents a radiological finding rather than a diagnosis [4]. Although it can occur as a primary disease, PI occurs more commonly secondary to other causes which range from benign conditions to fulminant disease [4,5]. Although the pathogenesis and etiology of PI are not fully understood, its causes can be classified into several conditions: life-threatening bowel necrosis, such as necrotizing enterocolitis and bowel ischemia; raised intra-abdominal pressure due to bowel obstruction, abdominal trauma, ileus, surgery and colonoscopy; increased mucosal permeability associated with steroid therapy, chemotherapy, connective tissue disease and immunosuppressive therapy; respiratory conditions such as chronic bronchitis, emphysema and asthma; trichloroethylene exposure, ingestion of carbohydrate such as lactose or sorbitol; and counter-perfusion super-saturation [4-7]. However, in the present case, no mechanical or bacterial factors, such as bacterial ischemia, bowel obstruction, inflammatory bowel disease, obstructive pulmonary disease or infectious colitis, were identified. In addition, the intraluminal air extended continuously from the ascending colon to the rectum, a pattern which is incompatible with any vascular territory, and an ischemic cause was therefore unlikely. The intra-abdominal metastases of lung adenocarcinoma showed a marked improvement in response to gefitinib therapy. However, the PI developed after the commencement of gefitinib and gradually resolved following its discontinuation. The patient was not taking any other medicines that might have been the cause of PI. We therefore presume that gefitinib played a major role in the development of PI in the present case.

Several chemotherapeutic agents have been reported to be associated with PI, including cyclophosphamide, methotrexate, vincristine, doxorubicin, daunorubicin, cytarabine, fluorouracil, paclitaxel, docetaxel, etoposide, irinotecan and cisplatin [8-10]. Since the intestinal mucosa is highly proliferative, chemotherapy often causes mucosal damage. Chemotherapeutic agents may also interfere with the mucosal integrity of the intestinal tract, resulting in extensive intramural air. However, PI secondary to molecularly-targeted agents is very rare. Recent reports describe the development of PI in patients receiving anti-vascular endothelial growth factor (VEGF) agents such as bevacizumab, sunitinib, and sorafenib [11-13]. VEGF inhibition can damage the microvasculature of the intestinal wall and it is plausible that a secondary insult to the intestine may lead to the development of PI. EGFR tyrosine kinase inhibitors such as gefitinib are associated with more gastrointestinal symptoms than other molecularly-targeted agents, partly as a result of their oral formulation. Although the precise pathophysiology of anti-EGFR agent-related gastrointestinal toxicity remains unclear, EGF is involved in the maintenance of mucosal integrity [14]. EGF deficiency secondary to anti-EGFR agents interferes with the mucosal integrity of the intestinal tract resulting in diarrhea, constipation, nausea and vomiting, and PI.

Furthermore, the patient described herein had a hepatic PVG on CT scan of the abdomen. The finding of PI and PVG during CT scanning usually indicates mesenteric ischemia or infarction [15,16]. However, both findings are also observed in a range of non-ischemic conditions. Therefore, conservative therapy may be effective, although immediate laparotomy is usually recommended in patients presenting with PVG [17]. In the present case, non-invasive management was indicated since the hepatic PVG was secondary to gefitinib-induced gastrointestinal toxicity rather than to bowel necrosis.

To our knowledge, the present report is the first to identify gefitinib as a cause of PI and PVG, and thus extends the literature concerning gefitinib-induced gastrointestinal toxicity. In conclusion, although PI and PVG are extremely rare complications of targeted molecular therapy, clinicians should be aware of the possibility of PI and PVG in patients receiving anti-EGFR therapies. Further studies are warranted to analyze the biological effects of targeted therapies on normal tissues as well as on cancer cells.

## Competing interests

The authors declare that they have no competing interests.

## Authors' contributions

LJY is the first author; HHS is the corresponding author of the manuscript. LOJ provided pathologic evaluation. LSN and SYK collected the patient's data and provided figures. CYH, LKH and KST were involved in drafting and revising the manuscript. The final version of the manuscript was seen and approved by all authors.

## Consent

Written informed consent for publication of this case report and associated images could not be obtained from the patient, but the patient's son provided the required consent.

## Pre-publication history

The pre-publication history for this paper can be accessed here:

http://www.biomedcentral.com/1471-2407/12/87/prepub
